# Bacterial Endotoxin Induces Oxidative Stress and Reduces Milk Protein Expression and Hypoxia in the Mouse Mammary Gland

**DOI:** 10.1155/2020/3894309

**Published:** 2020-03-24

**Authors:** Alexander Jonathan Spitzer, Qing Tian, Ratan K. Choudhary, Feng-Qi Zhao

**Affiliations:** Department of Animal and Veterinary Sciences, University of Vermont, 570 Main Street, Burlington, VT 05452, USA

## Abstract

The aim of this study was to investigate the mechanisms underlying the reduced milk production during mastitis. We hypothesized that bacterial endotoxin induces hypoxia, oxidative stress, and cell apoptosis while inhibiting milk gene expression in the mammary gland. To test this hypothesis, the left and right sides of the 4^th^ pair of mouse mammary glands were alternatively injected with either lipopolysaccharide (LPS, *E. coli* 055: B5, 100 *μ*L of 0.2 mg/mL) or sterile PBS through the teat meatus 3 days postpartum. At 10.5 and 22.5 h postinjection, pimonidazole HCl, a hypoxyprobe, was injected intraperitoneally. At 12 or 24 h after the LPS injection, the 4^th^ glands were individually collected (*n* = 8) and analyzed. LPS treatment induced mammary inflammation at both 12 and 24 h but promoted cell apoptosis only at 12 h. Consistently, H_2_O_2_ content was increased at 12 h (*P* < 0.01), but dropped dramatically at 24 h (*P* < 0.01) in the LPS-treated gland. Nevertheless, the total antioxidative capacity in tissue tended to be decreased by LPS at both 12 and 24 h (*P* = 0.07 and 0.06, respectively). In agreement with these findings, LPS increased or tended to increase the mRNA expression of antioxidative genes *Nqo1* at 12 h (*P* = 0.05) and *SLC7A11* at 24 h (*P* = 0.08). In addition, LPS inhibited mammary expression of *Csn2* and *Lalba* across time and protein expression of *Csn1s1* at 24 h (*P* < 0.05). Furthermore, hypoxyprobe staining intensity was greater in the alveoli of the PBS-treated gland than the LPS-treated gland at both 12 and 24 h, demonstrating a rise in oxygen tension by LPS treatment. In summary, our observations indicated that while intramammary LPS challenge incurs inflammation, it induces oxidative stress, increases cell apoptosis and oxygen tension, and differentially inhibits the milk protein expression in the mammary gland.

## 1. Introduction

The major burden costing the dairy industry is mastitis, the inflammation of the mammary gland caused by bacterial infection. The major contributor to the loss is reduced milk production [1, 2]. Mastitis comes in many forms, from acute clinical mastitis to chronic subclinical mastitis, as it is caused by a plethora of bacteria. Clinical mastitis causes the mammary gland to become swollen, red, and hot with vasodilation and is often painful for the mother, whereas subclinical mastitis is often asymptomatic but still ends with reduced milk yield [1]. During mastitis, the immune cell recruitment to the site of infection enhances the somatic cell count (SCC), and broken mammary epithelial cell (MEC) tight junctions increase sodium content in the milk [3]. The milk SCC and sodium content usually increase with severity of mastitis as the milk yield decreases, leading to reduced milk quality. Mastitis not only causes acute effects on the cow but also presents chronic issues as milk production in cattle and goats with resolved mastitis is lower than unafflicted cows and goats [4, 5]. In subclinical mastitis, the development of antibiotic resistance adds on costs for veterinary care and premature culling [6, 7].

The type of infection is in part dependent on the etiological agent. Subclinical mastitis is most often caused by Gram-positive bacteria whereas Gram-negative bacteria commonly cause acute clinical mastitis [8]. Common Gram-positive bacteria found to cause mastitis include the infamous *Staphylococcus aureus* (*S. aureus*), *Streptococcus agalactiae* (*S. agalactiae*), and *Streptococcus uberis* (*S. uberis*). These pathogens are spread mostly by physical contact with carrying animals and people, which is why it is considered contagious mastitis. Gram-negative bacteria, on the other hand, are usually spread through contact with fecal matter. These bacteria are often pathogenic strains of *Enterobacteriaceae* found in mammalian gastrointestinal tracts, such as *Escherichia coli* (*E. coli*). These bacteria are not as well adept at evading the host immune system as Gram-positive bacteria; thus, they are targeted for destruction in the host. The lysis of these bacteria by soluble factors like *β*-defensin releases endotoxin lipopolysaccharide (LPS), a superantigen that overstimulates the host immune system to cause acute inflammation of the mammary gland, endotoxemic shock, and other symptoms seen in clinical mastitis [9, 10]. Heat-inactivated *E. coli* have been shown to activate proinflammatory signaling more strongly than heat-inactivated *S. aureus* [9].

It is well known that bacterial infection initiates a cascade of innate immune responses. Macrophages and MECs work in concert to recruit polymorphonuclear neutrophils (PMNs) to the tissue, which help combat the pathogen via phagocytosis and reactive oxygen species- (ROS-) mediated destruction in addition to cytokine production that supports a proinflammatory and antimicrobial environment [11]. These actions that resolve the infections are not without costs as the tight junctions of MECs loosen as a result of proinflammatory signaling and PMN diapedesis [12, 13]. Some of the MECs undergo apoptosis because of these stresses which induce caspase 3 expression or activation [14, 15]. Because of the dramatic increase in PMN number recruited into the infected areas, we hypothesized that mastitis may cause localized hypoxia due to increased oxygen consumption by these cells.

The massive leukocyte recruitment can also increase the local free radical production to unbalanced levels, a pathological state known as oxidative stress, and stimulate antioxidative response. Rising reactive oxygen species (ROS) were observed in rodent mammary glands after LPS challenge [16, 17]. The antioxidative response takes on oxidative stress at multiple levels. Soluble factors, such as glutathione or vitamin E, help to immediately resolve oxidative stress, but these factors are decreased as oxidative stress increases [18]. To restore the balance, the nuclear factor erythroid 2-related factor 2- (Nrf2-) antioxidant responsive element (ARE) pathway may be activated to stimulate the synthesis of antioxidant enzymes, such as haemoxygenase-1 (Hox1) and NAD(P)H quinone oxidoreductase 1 (Nqo1) [19]. These factors can work together to reduce the oxidant content and reestablish the redox balance.

Because of its large economic impact to dairy industry, mastitis research has become an increasingly progressive field as many look to the pathology of this disease and find therapeutic treatments to combat it. The most common approach to study mastitis has been through an intramammary infusion of proinflammatory bacteria toxin into the mammary gland using bovine and rodent models [12, 20, 21]. While they do not exactly mimic bovine lactation, mice and rats have widely been used for mastitis models for over 40 years because of their advantages of easier maintenance and cost effectiveness compared to bovine counterpart [22]. Whereas most of these studies have examined the effects of potential therapeutics on inflammation, oxidative stress, and apoptosis in the mammary gland, limited studies have investigated the effects of bacterial toxins on milk protein expression, and no study to our knowledge has examined the oxygen tension in the mammary gland during mastitis.

The objective of this study was to investigate the time-dependent effects of bacterial LPS challenge on oxidative stress, oxygen tension, and expression of milk protein genes and genes involved in cell apoptosis and antioxidation in the mammary gland using a mouse model with a unilateral design.

## 2. Materials and Methods

### 2.1. Animal Treatment and Tissue Sample Collection

Sixteen 8-week-old female BALB/cJ mice (Jackson Labs, Bar Harbor, ME, USA) were used for this study, and all procedures of animal use were approved by the University of Vermont Institutional Animal Care and Use Committee (IACUC Protocol #17-030). Mice were kept in breeding cages in harems of 1 male and 2 females under a controlled environment (25°C, 45% humidity and 12 h light-dark cycle) and were bred to pregnancy. Three days postpartum, all lactating mice were anesthetized with 4.0% isoflurane and then received an intramammary infusion (IMI) of either LPS (*E. coli* 055: B5, #6529, Sigma, St. Louis, MO, USA; 100 *μ*L of 0.2 mg/mL) or sterile phosphate-buffered saline (PBS, 137 mM NaCl, 2.7 mM KCl, 8 mM Na_2_HPO_4_, and 2 mM KH_2_PO_4_, pH 7.4; 100 *μ*L), alternatively into either side of the 4^th^ mammary glands through the teat meatus with 30G 0.5 mL insulin syringes (Becton, Dickinson & Company, Franklin Lakes, NJ, USA), and the injection site was thoroughly and gently massaged. At 10.5 and 22.5 h post-IMI, 1.5 mg/mL pimonidazole HCl (#HP2-1000 kit, Hypoxyprobe, Burlington, MA, USA) in sterile water was injected intraperitoneally (*i.p.*) into each mouse. At 12 or 24 h after the LPS infusion, eight mice were euthanized by cervical dislocation to maintain oxygen tension in the microenvironments of the tissues. The 4^th^ glands were individually collected immediately from all animals (*n* = 8), and tissue samples were either snap-frozen in liquid nitrogen or later stored in -80°C or chemically fixed as described below.

### 2.2. RNA Isolation, Reverse Transcription (RT), and Quantitative Real-Time Polymerase Chain Reaction (qPCR)

Frozen mammary tissue was weighed to 30 mg, dispersed, and ground by mortar and pestle chilled in liquid nitrogen. Ground tissue was placed directly into 600 *μ*L of lysis buffer (RLT, Qiagen, Venlo, Netherlands) with 1% *β*-mercaptoethanol (Sigma) and was homogenized by an electric homogenizer in 3 × 10s bursts with 10s in ice between bursts to avoid rise in temperature. Total RNA was then isolated using RNEasy Mini Kit (#74104, Qiagen) according to the manufacturer's instruction. Total RNA was treated with DNase I (#79254, Qiagen) and eluted in ultrapure water. The RNA concentration and quality were evaluated by Nanodrop 2000 spectrophotometric analyzer (#ND-2000, Thermo Fisher Scientific, Waltham, MA, USA), and RNA quality was further analyzed by Agilent 2100 Bioanalyzer (Agilent, Santa Clara, CA, USA) to verify RNA integrity number values ≥ 8.0.

cDNA synthesis was performed using Invitrogen SuperScript III cDNA Synthesis Kit (#18080051, Invitrogen, Waltham, MA, USA) according to manufacturer's specifications in an Applied Biosystems 2720 Thermocycler (#4359659; Applied Biosciences, Waltham, MA, USA). RNA samples were diluted to 2 *μ*g for 8 *μ*L reaction with ultrapure grade water with 1 *μ*L each of provided dNTP mix and Oligo (dT-20). Samples were denatured at 85°C for 5 min and then added with 10 *μ*L of SuperScript III reaction mix. Reactions were performed at 50°C for 50 min, 85°C for 5 min, and 4°C for 5 min. Reactions were collected by brief centrifugation for 1 min at 18,000 x g and digested with 1 *μ*L RNase H at 37°C for 20 min before stored at -20°C.

mRNA expression was determined by qPCR using iTaq SYBR Green Supermix (#1725121, Bio-Rad, Hercules, CA, USA). 1 *μ*L of cDNA solution was mixed with 500 nM forward and reverse primers (Table 1) in 10 *μ*L, and 10 *μ*L of iTaq SYBR Green Supermix was added to each reaction. The reactions were carried out in Bio-Rad CFX96 Thermocycler using the following program: initial denaturation at 95°C for 30 s and 40 cycles of 5 s at 95°C for denaturation and 30 s at 60°C for annealing and extension followed by melt curve analysis from 65 to 95°C at 0.5°C increments at every 5 s. The qPCR data was analyzed by 2^-*ΔΔ*Ct^ method [23] and normalized by the expression levels of the housekeeping genes *ActB*, *Hprt*, *Gapdh*, *Stx5a*, and *Hnrnpab*.

### 2.3. Protein Isolation and Western Blot

200 mg of frozen mouse mammary tissue was lysed in 1,500 *μ*L NP-40 lysis buffer (#FNN0021, Fisher, Waltham, MA, USA) with protease inhibitor cocktail (#8340, Sigma) and pulverized by Dounce homogenizer at 4°C. Whole tissue lysates were then agitated at 4°C for 2 h and centrifuged at 13,400 x g for 20 min at 4°C. Supernatants were divided into aliquots and stored at -80°C. Concentration of tissue lysates was determined by the Bradford Colorimetric assay (#5000001, Bio-Rad) with bovine serum albumin (BSA) protein standard (#500-0007, Bio-Rad). Based on these concentrations, tissue lysates were diluted in lysis buffer to achieve a volume of 300 *μ*L before addition of 100 *μ*L 4× Laemmli buffer [62.5 mM Tris-HCl, pH 6.8, 10% glycerol, 2% sodium dodecyl sulfate (SDS), and 0.00125% (weight/volume) bromophenol blue] for a final protein concentration of 3.33 *μ*g/*μ*L. Samples were vortexed and immediately placed in 100°C heat block for 10 min.

15 *μ*L samples were loaded into gels for SDS-polyacrylamide gel electrophoresis (SDS-PAGE) (stacking gel: 0.125 M Tris-HCl, pH 6.8, 0.05% ammonium persulfate, 0.1% TEMED, and 4% SDS-polyacrylamide; separating gel: 1.5 M Tris-HCl, pH 8.3, 0.05% ammonium persulfate, 0.05% TEMED, and 12% SDS-polyacrylamide) and ran at 150 V in electrode buffer (1.5% Tris base, pH 8.3, 7.2% glycine, and 0.5% SDS). The separated proteins were transferred to polyvinylidene fluoride membranes at 95 V and 4°C for 90 min with transfer buffer (25 mM Tris, pH 8.3, 192 mM glycine, and 20% methanol), and the blots were stained with 1% Ponceau S solution to evaluate protein transfer efficiency. Blots were blocked in 5% BSA in Tris-buffered saline with 0.1% Tween-20 (TBS-T, #P1379, Sigma).

Blots were incubated overnight on a plate shaker at 4°C with primary antibody for *β*-casein (#sc-166684, Santa Cruz Biotechnology, Santa Cruz, CA, USA; 0.2 *μ*g/mL), *α*-S1-casein (#sc-365929, Santa Cruz Biotechnology; 0.2 *μ*g/mL), *α*-lactalbumin (#abx101423, Abbexa, Cambridge Science Park, Cambridge, United Kingdom; 0.2 *μ*g/mL), and cleaved caspase 3 (CC3; #CST-9661, Cell Signaling, Danvers, MA, USA; 1 : 1000). Blots were washed 3 times with TBS-T for 10 min intervals followed by incubation with secondary antibodies [goat horseradish peroxidase- (HRP-) linked antirabbit IgG: #CST-7074P2, Cell Signaling; goat HRP-linked antimouse IgG: #sc-2005, Santa Cruz Biotechnology] at 1: 10,000 dilution and room temperature for 1 h. Blots were then washed 4 times in TBS in 10 min intervals before incubation with ECL-substrate solutions (#34577, Invitrogen). Blots were then imaged with ChemiDoc ECL Imager (Bio-Rad), stripped for 15 min in Restore Western Blot Stripping Buffer (#21063, Invitrogen), and reprobed with antibody for GAPDH (#2118, Cell Signaling; 1 : 5000). Optical density of images was evaluated by ImageJ (ver. 5.12a; NIH, Bethesda, MD, USA) [24] and normalized by the expression of GAPDH.

### 2.4. Histological Staining and Immunohistochemistry

Fresh mouse mammary tissue was cut into 3-5mm pieces and fixed in neutral buffered PBS with 10% formalin (#HT501128, Sigma) at room temperature for 4-6 h and stored in 70% ethanol at room temperature. Tissue embedding, deparaffinization, sectioning, and hematoxylin and eosin staining were performed by the Pathology Department of the University of Vermont Medical Center (Burlington, VT, USA). For immunostaining, 5-*μ*m-thick tissue sections were deparaffinized in xylene (3 × 5 min) followed by dehydration in absolute ethanol (2 × 3 min) and gradual rehydration in 95% ethanol (2 × 3 min), 70% ethanol, and deionized water (2 × 2 min). Antigen retrieval was done on hot plate for 10 min in boiling citrate buffer at pH 6.0 (Vector lab, Burlingame, CA, USA) followed by 30 min of cooling at room temperature. Slides were washed in PBST (PBS with 0.5% of tween 20). After blocking nonspecific protein binding with 2.5% horse serum (Vector Lab) for 20 min, slides were incubated with FITC conjugated anti-pimonidazole mouse IgG1 monoclonal antibody (1 : 100 dilution in 2.5% horse serum; Hypoxyprobe™ Plus Kit, Hypoxyprobe, Inc.) for 1.5 h at room temperature in moist chamber. Slides were washed with PBST (3 × 2 min) before mounting with Vectashield with 4,6-diamidino-2-phenyl-indole (DAPI) (Vector Lab). Slides were viewed with a fluorescence microscope (model Eclipse 50xi, Nikon Instruments Inc., NY, USA) at 200× magnification under green (FITC) and blue (DAPI) channels.

5-7 images/slides were captured from LPS- and PBS-treated glands under constant illumination. Images were opened in ImageJ, and inner and outer boundaries of alveoli were marked using freeform drawing tool. From each image, 12 distinct and intact alveoli were marked and measured for area, integrated density, standard deviation, and mean gray value of hypoxia signal using a freeform drawing tool. The difference in raw integrated intensity of outer and inner boundary of alveoli provided the mean integrated intensity of hypoxia probe signal of mammary epithelium.

### 2.5. Oxidative Stress and Antioxidation Assays

Hydrogen peroxide (H_2_O_2_) levels in mammary tissues were analyzed using Sigma Fluorimetric Hydrogen Peroxide Assay Kit (#MAK166) according to manufacturer's specifications. Briefly, protein lysates from Western blot analysis were diluted to 1 mg/mL in NP-40 lysis buffer without protease inhibitors to avoid downstream interference of the assay. In 96-well plates, 50 *μ*L of each samples and standards of 0, 0.1, 0.3, 1, 3, and 10 *μ*M H_2_O_2_ was laid out, and each reaction was mixed with 50 *μ*L reaction mixture consisting of assay buffer with 20 units/mL horseradish peroxidase and 1% infrared fluorometric peroxidase substrate. Plates were incubated at room temperature (23°C) for 10 min and then read fluorometrically at 640 nm excitation/680 nm emission on a H4 Plate Reader (Biotek Synergy, Winooski, VT, USA).

Mammary gland total antioxidant capacity was analyzed using Sigma Antioxidant Assay Kit (#CS0790) according to manufacturer's specifications. Approximately 100 mg tissue was lysed in Dounce homogenizer in assay buffer at 4°C. Lysates were centrifuged at 12,800 x g at 4°C for 15 min, and the supernatants were collected and aliquoted. For each reaction, 10 *μ*L of 10 *μ*g/mL tissue lysate was mixed with 10 *μ*L horse myoglobin in ultrapure water. Standard curve was prepared with (±)-6-hydroxy-2,5,7,8-tetramethylchromane-2-carboxylic acid (Trolox) at 0, 0.015, 0.045, 0.105, 0.21, and 0.42 mM, respectively. Before adding reaction buffer, 25 *μ*L 3% H_2_O_2_ was mixed with 10 mL ABTS buffer to activate the reagent. To each reaction in a 96-well plate, 150 *μ*L ABTS reaction buffer with H_2_O_2_ was added to reactions and incubated for 5 min at room temperature (23°C). 100 *μ*L stop buffer was added, and the plate was read for optical density at 405 nm in a Biotek Synergy 2 Plate Reader. The total antioxidant capacity of tissue samples was derived from the standard curve.

### 2.6. Statistical Analysis

All the data were analyzed by two-way mixed ANOVA using PROC MIXED model in SAS (ver. 9.4; SAS Institute, Cary, NC, USA). Time (12 and 24 h) and treatments (LPS and PBS) were fixed factors each at two levels. The treatment was considered a repeated measure, whereas time was not repeated. Effects of animal and side of gland (left or right) were considered as random effects. Differences in means of each variable were detected by type 3 tests for fixed effects (treatment and time) and interactions (time∗treatment). Data are expressed as mean ± SEM, and the differences between means were declared significant (*P* < 0.05) or with a tendency of significance (0.05 < *P* < 0.10).

## 3. Results

### 3.1. LPS Challenge Incurred Mammary Gland Inflammation

**Figure 1:**
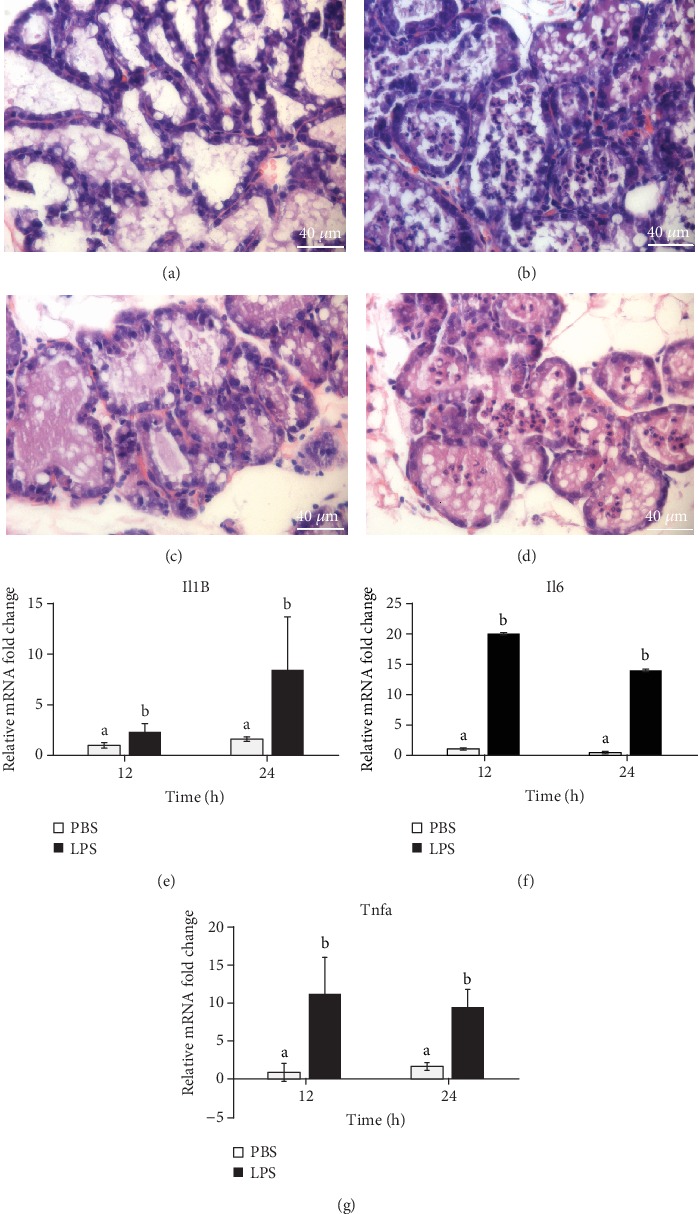
Lipopolysaccharide (LPS) challenge incurs mammary gland inflammation. The left and right sides of the 4^th^ mammary glands were alternatively injected with LPS or PBS through teats at day 3 of lactation. The injected glands were individually collected after 12 or 24 h of injection (*n* = 8). Hematoxylin and eosin staining of formalin-fixed, paraffin-embedded mammary tissue sections of 12 h ((a) PBS and (b) LPS) and 24 h ((c) PBS and (d) LPS). mRNA expression of cytokines (e) interleukin-1*β* (*Il1B*), (f) interleukin-6 (*Il6*), and (g) tumor necrosis factor-*α* (*Tnfa*) by real-time quantitative PCR. Relative gene expression of cytokines was calculated by the 2^-*ΔΔ*Ct^ method and normalized by the mRNA levels of the internal control housekeeping genes *Actb*, *Gapdh*, *Hrpt*, *Stx5a*, and *Hnrnpab*. Error bars represent standard error, and different letters above each bar indicate significant differences. Significance was declared when *P* < 0.05.

Hematoxylin and eosin staining of tissue sections showed that at 12 h of treatment, intramammary challenge of LPS induced massive recruitment of cells, likely PMN based on cell morphology [25], into alveoli compared to PBS challenge (Figures 1(a) and 1(b)), and this recruitment was also evident at 24 h of LPS treatment (Figures 1(c) and 1(d)). Moreover, mRNA expression of cytokines IL-1B, IL-6, and TNF-*α* was dramatically induced in the mammary gland by LPS at both 12 and 24 h (*P* < 0.05), but no difference was seen across time (Figures 1(e)–1(g)).

### 3.2. LPS Induced Mammary Apoptosis

**Figure 2:**
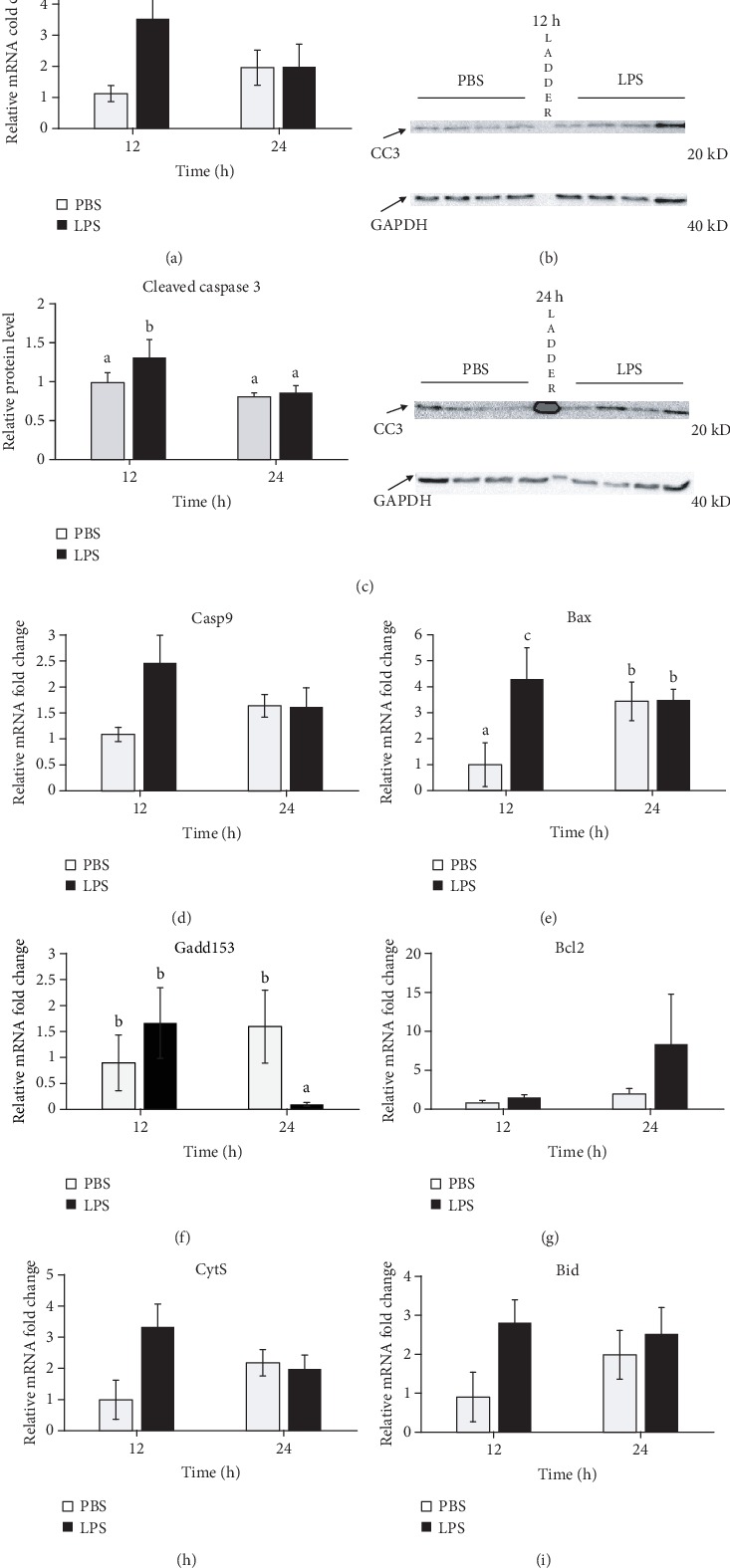
Lipopolysaccharide (LPS) treatment induced apoptosis in the mammary gland. Mammary tissues treated with PBS or LPS for either 12 or 24 h (*n* = 8) were collected and analyzed by real-time PCR for (a and d–i) mRNA expression or Western blot for protein levels ((b) representative blots in which each lane was a tissue sample from a different animal except the protein size ladder in the middle lane and (c) quantitative representation). (a) Caspase 3 (*Casp3*), (b and c) cleaved caspase 3 (CC3), (d) caspase 9 (*Casp9*), (e) Bcl-2-associated protein X (*Bax*), (f) endoplasmic reticulum stress marker C/EBP homologous protein (CHOP; *Gadd153*), (g) B-cell lymphoma 2 (*Bcl2*), (h) cytochrome-C (*CytS*), and (i) BH3-interacting domain (*Bid*). Relative mRNA expression was calculated by the 2^-*ΔΔ*Ct^ method and normalized by expression levels of housekeeping genes *Actb*, *Gapdh*, *Hrpt*, *Stx5a*, and *Hnrnpab*. Protein expression of CC3 was normalized by GAPDH. Error bars represent standard error, and different letters above each bar indicate significant differences. Significance was declared when *P* < 0.05.

The effect of LPS on mammary gland apoptosis was investigated by the analyses of proapoptotic marker expression. Compared to PBS treatment, LPS treatment did not significantly increase mRNA expression of both caspase 3 and caspase 9 at 12 h and 24 h (Figures 2(a) and 2(d)), but the protein level of CC3 was significantly increased (1.33-fold) by LPS treatment at 12 h (*P* = 0.02, Figures 2(b) and 2(c)). In accordance with these findings, LPS treatment increased the mRNA expression of proapoptotic factor Bax 4.3-fold at 12 h (*P* = 0.01), but not at 24 h (Figure 2(e)). However, expression of the endoplasmic reticulum (ER) stress marker Chop was not significantly changed by LPS treatment at 12 h but was decreased dramatically by LPS treatment at 24 h (*P* = 0.01, Figure 2(f)). Expression of Bax and Chop presented an overall interaction between time and treatment (*P* < 0.05). No significant differences were observed in mRNA expression of *Bcl2*, *CytS*, and *Bid* between the two treatments at both time points (Figures 2(g)–2(i)).

### 3.3. LPS Induced Oxidative Stress and Stimulated the Antioxidative Response in the Mammary Gland

**Figure 3:**
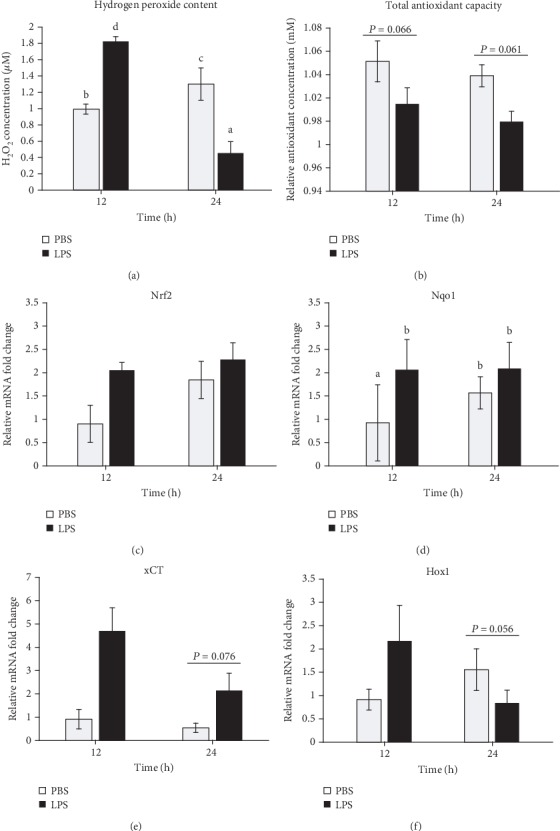
Lipopolysaccharide (LPS) increases oxidative stress and activates the antioxidative response in the mammary gland. Mammary tissues treated with PBS or LPS for either 12 or 24 h (*n* = 8) were collected and analyzed for the (a) concentration of hydrogen peroxide, (b) total antioxidant capacity relative to antioxidant standard Trolox, and (c) mRNA expression of nuclear factor erythroid 2-related factor 2 (Nrf2), (d) NAD(P)H quinone oxidase 1 (Nqo1), (e) cysteine transporter xCT, and (g) heme oxygenase 1 (Hox1). Gene expression was calculated by the 2^-*ΔΔ*Ct^ method and normalized by the levels of housekeeping genes *Actb*, *Gapdh*, *Hrpt*, *Stx5a*, and *Hnrnpab*. Error bars represent standard error, and different letters above each bar indicate significant differences. Significance was declared when *P* < 0.05.

LPS treatment induced oxidative stress in the mammary gland at 12 h, demonstrated by a significant increase (1.8-fold) in tissue H_2_O_2_ levels compared to PBS treatment (*P* < 0.01, Figure 3(a)); however, the tissue H_2_O_2_ level was decreased by LPS at 24 h (*P* < 0.01). The total antioxidative capacity (TAC) of the mammary gland tended to be lower at both 12 (*P* = 0.07) and 24 h (*P* = 0.06) of LPS treatment (Figure 3(b)). These dynamics demonstrated an interaction between time and treatment (*P* < 0.05).

LPS challenge did not significantly change the mRNA expression of Nrf2, a master regulator of the antioxidative response, in the mammary gland at both 12 and 24 h (Figure 3(c)). However, LPS treatment significantly increased mRNA expression of Nrf2 target gene Nqo1 at 12 h (*P* = 0.05, Figure 3(d)) and tended to increase mRNA expression of cystine transporter xCt at 24 h (*P* = 0.08, Figure 3(e)). However, LPS treatment tended to decrease mRNA expression of Hox1 genes, including Nqo1 at 24 h (*P* = 0.06, Figure 3(f)). Expression of Nqo1 and Hox1 showed significant interactions between time and treatment (*P* < 0.05).

### 3.4. LPS Treatment Reduced Milk Protein Gene Expression

**Figure 4:**
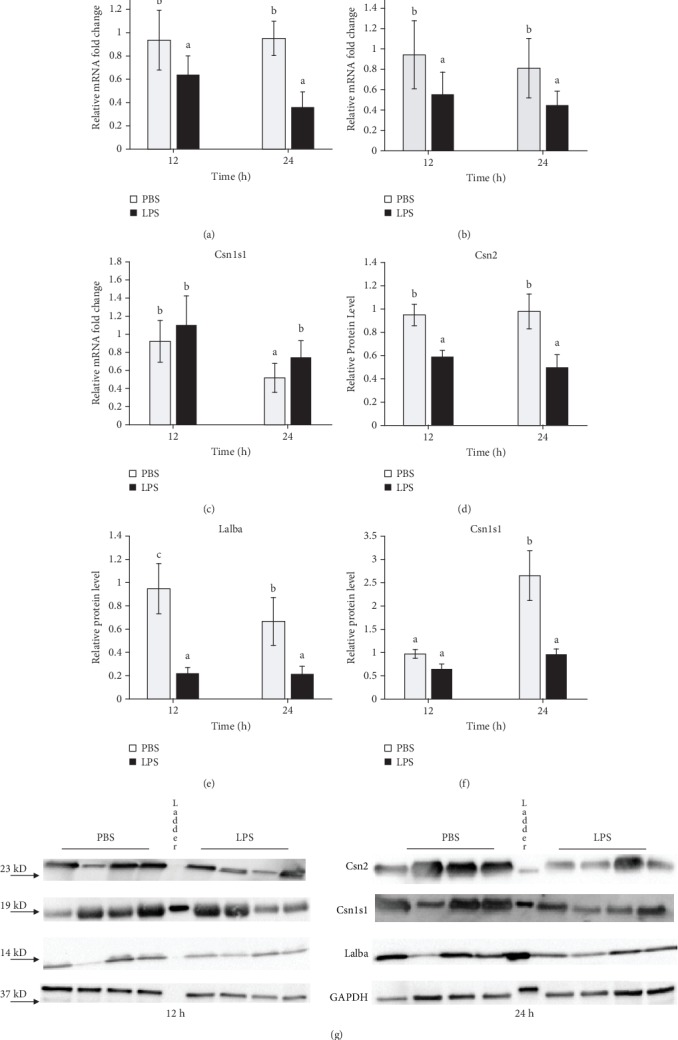
Lipopolysaccharide (LPS) challenge reduces milk protein expression in the mammary gland. Mammary tissues treated with PBS or LPS for 12 or 24 h (*n* = 8) were collected and analyzed by real-time PCR for (a–c) mRNA expression and Western blot for protein levels ((d–f) quantitative presentation and (g) representative images in which each lane was a tissue sample from a different animal except the protein size ladder in the middle lane) of (a and d) *β*-casein (*Csn2*), (b and e) *α*-lactalbumin (*Lalba*), and (c and f) *α*-S1-casein (*Csn1s1*). Relative mRNA expression was calculated by the 2^-*ΔΔ*Ct^ method and normalized by the expression of housekeeping genes *Actb*, *Gapdh*, *Hrpt*, *Stx5a*, and *Hnrnpab*. In (d)–(f), relative protein levels of milk proteins were normalized by the levels of GAPDH (d). Error bars represent standard error, and different letters above each bar indicate significant differences. Significance was declared when *P* < 0.05.

Compared to the PBS-treated glands, mRNA expression of milk protein genes *Csn2* and *Lalba* was significantly decreased in LPS-treated glands at both 12 h (68% and 59% of control, respectively; *P* < 0.05) and 24 h (39% and 47% of control, respectively; *P* < 0.05) (Figures 4(a) and 4(b)). In contrast to *Csn2* and *Lalba*, *Csn1s1* mRNA expression was not affected at 12 h but was significantly increased by LPS treatment at 24 h (*P* = 0.01) (Figure 4(c)).

At the protein level, expression of all three major milk proteins in the mammary gland was significantly inhibited by LPS treatment at 12 and 24 h (*P* < 0.05), except that no effect was seen for CSN1S1 at 12 h (Figures 4(d)–4(g)). CSN1S1 protein level showed an overall interaction between time and treatment (*P* < 0.05).

### 3.5. LPS Treatment Increased Oxygen Tension in the Mammary Gland

**Figure 5:**
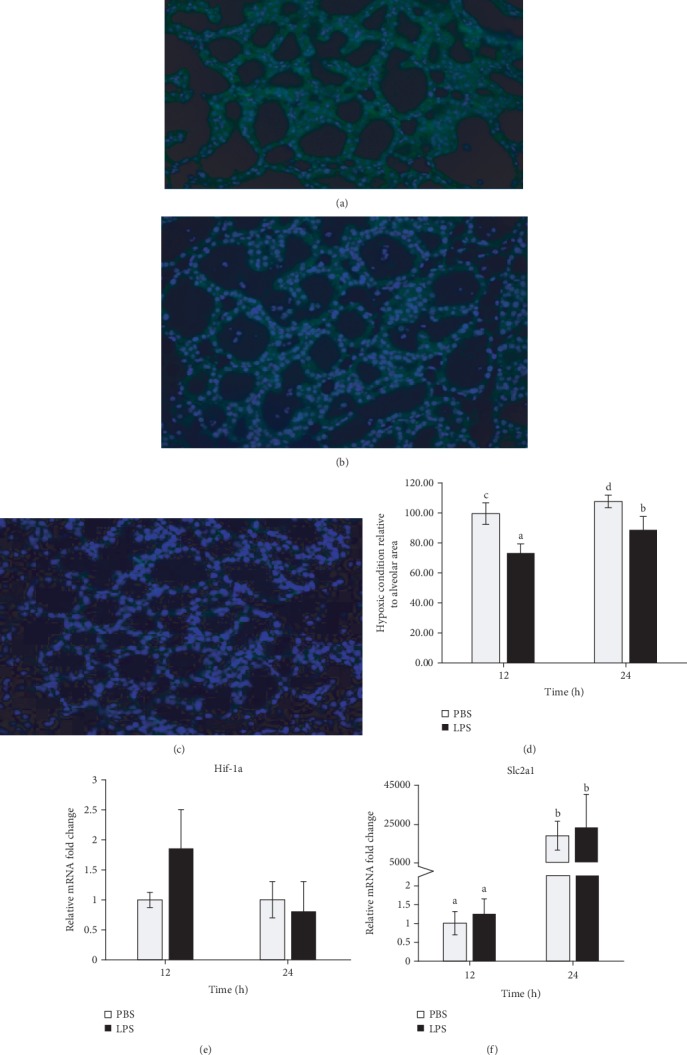
Effect of Lipopolysaccharide (LPS) treatment on hypoxic condition in the mammary gland. Mice were injected intraperitoneally with pimonidazole HCl, and mammary tissues were treated with PBS or LPS for 12 or 24 h (*n* = 8). (a–c) Representative immunostaining of hydroxyprobe in the (a) PBS-treated gland and (b) LPS-treated gland at 12 h. (c) Negative control staining performed by the omission of the first antibody against hypoxyprobe. (d) Quantitative representation of relative hypoxic condition in LPS- and PBS-infused glands. mRNA expression of (e) hypoxia-inducible factor 1 (*Hif1a*) and (f) glucose transporter 1 (*Slc2a1*) by real-time PCR. Relative gene expression was calculated by the 2^-*ΔΔ*Ct^ method and normalized by the expression levels of housekeeping genes *Actb*, *Gapdh*, *Hrpt*, *Stx5a*, and *Hnrnpab*. Error bars represent standard error, and different letters above each bar indicate significant differences. Significance was declared when *P* < 0.05.

To investigate whether LPS treatment induces hypoxic condition in the mammary gland, we injected LPS-treated mice with the hypoxyprobe pimonidazole HCl which binds to amino acids and proteins in hypoxic conditions. To our surprise, fluorescent staining using FITC conjugated specific antibody to pimonidazole adducts in hypoxic cells showed that LPS treatment relieved hypoxic conditions in the mammary gland at both 12 and 24 h (*P* < 0.05) (Figures 5(a)–5(d)). Despite this, mRNA expression of *Hif1a* and *Slc2a1* did not change significantly with LPS infusion although *Slc2a1* expression increased drastically at 24 h compared to 12 h (Figures 5(e) and 5(f)).

## 4. Discussion

The overall objective of this study was to investigate how mastitis causes a reduction in milk production. To approach this goal, we studied the time-dependent effects of the major bacterial endotoxin LPS on inflammation, apoptosis, oxidative stress, oxygen tension, and milk protein gene expression in the mammary gland using a mouse model. In this model, the 4^th^ pair of the mammary glands were unilaterally challenged with either LPS or PBS through the teats. A major advantage of this model is the higher power in statistical analysis due to the use of both treatment and control in the same animal [26], whereas a potential weakness of this model is the possibility of presence of a systemic effect of LPS treatment. Although we cannot rule out the possibility that LPS injected to one gland might enter the blood stream and travel to the PBS-injected gland to cause effects, these effects should be minimal because cytokine expression and PMN recruitment were much lower in PBS gland vs LPS gland.

As expected, intramammary LPS challenge in this study incurred mammary inflammation, demonstrated by a large increase of PMN infiltration into the alveolar lumen. Recruitment of PMN by LPS has been well shown in bovine and mouse mammary glands in previous studies [11, 25]. This recruitment results in a large increase in somatic cell count (SCC) in milk and is induced by inflammatory mediators, including cytokines IL-1B, IL-6, and TNF-*α*, from macrophages and MECs [11, 25, 27, 28]. The surge of expression of cytokines in the mammary gland by LPS challenge is also confirmed in this study.

In this study, mammary infusion of LPS significantly induced protein abundance of CC3 at 12 h, but not at 24 h, indicating a transient induction in cell apoptosis. Caspase 3 is a key death protease which plays a central role in the execution phase of cell apoptosis by catalyzing the specific cleavage of many key cellular proteins [29]. This enzyme is activated in the apoptotic cells both by death ligand-mediated and mitochondria-mediated pathways, and the activation of this enzyme requires proteolytic processing at conserved aspartic residues to produce two subunits [30]. The cleaved active form of caspase 3 is a widely used marker of cell apoptosis. In supporting caspase 3 activation, mRNA expression of another popular cell apoptosis marker gene *Bax* showed the similar changes by LPS in this study. Bax forms a heterodimer with BCL2 to activate cell apoptosis [31]. These observations were consistent with the observations from a previous study in which intramammary infusion of LPS increased Bax expression at the mRNA and protein levels and caspase 3 cleavage at 12 h, but not caspase 3 gene expression [32]. Induction of cell apoptosis by LPS were also observed by TUNEL assay in mouse and bovine mammary tissue treated with LPS [32, 33].

In this study, mRNA expression of an ER stress marker, Chop, was not increased at 12 h but was decreased at 24 h following LPS challenge. This observation was in contrast to increased Chop expression in bovine mammary epithelial cells after LPS treatment *in vitro* [34]. Chop is a primary signaling proteins behind ER stress-induced apoptosis when the unfolded protein response is unable to resolve the source of stress [35]. The Chop depression at 24 h may suggest a dramatic improvement of anti-ER stress responses or a decrease of ER stress after 24 h. This observation also supports possible decease in cell apoptosis at 24 h in LPS gland.

Consistent with CC3 expression, LPS induced a temporary increase in oxidative stress in the mammary gland at 12 h, demonstrated by increased H_2_O_2_ level. Rising ROS levels were also observed in rodent mammary gland after LPS challenge [16, 17]. Hydrogen peroxide is a natural metabolite and a signaling molecule in many organisms, but its overproduction by inflammatory and vascular cells during pathological conditions or over accumulation due to decreased antioxidation capacity can induce oxidative stress [36]. Milk from cattle with subclinical mastitis contains pronounced H_2_O_2_ content [37]. Hydrogen peroxide is the product of superoxide and hydroxyl radical breakdown by enzymes like superoxide dismutase 1 (Sod1) and Nqo1, whose activities are increased by circulating LPS [38]. The accumulation of ROS can have destructive effects on cellular proteins, carbohydrates, lipids, and DNA to impair their functions and initiate apoptosis. In fact, it is widely documented that H_2_O_2_ can stimulate cell death *in vitro* by activating p53/Bax/Caspase 3 proapoptotic pathways in bovine MECs [39, 40].

However, the H_2_O_2_ level was largely decreased at 24 h of LPS treatment, suggesting a release and improvement of oxidative stress. This decrease was likely due to decreased production of ROS at this time because the TAC in the mammary gland was decreased at both times after LPS challenge. This is also supported by our histological analysis which indicated a reduced lymphatic cell infiltration, a major source of H_2_O_2_, in the mammary gland, and was consistent with the Chop expression. The reduction of TAC by LPS or mastitis has been shown in previous studies. For example, cattle with subclinical mastitis showed lowered TAC with increased SCC in milk [37]. Even increased LPS content in the plasma significantly lowered the TAC of mammary tissue of cattle experiencing subacute ruminal acidosis incurred by a high-concentrate diet [38].

Nrf2 is a key regulator of antioxidation. During oxidative stress, it is activated by releasing from its binding with KEAP1 and then entering the nucleus to bind to the ARE in the upstream promoter of many antioxidative genes, including *Nqo1*, *Slc7a11*, *Hox1*, and *Txnrd1* [41]. To study its potential role in mastitis, we analyzed its expression as well as expression of its several target genes in this study. LPS did not change Nrf2 mRNA levels but upregulated the expression of Nqo1 at 12 h and tended to increase the expression of xCT at 24 h. Nqo1 is an enzyme which breakdown ROS into water and oxygen [42]. The cysteine/cystine transporter xCt is required for the synthesis of antioxidant glutathione. Taken together, our observations indicated a possible activation of Nrf2-ARE pathway by LPS.

In this study, mRNA or protein expression of Csn2 and Lalba were significantly decreased at 12 and 24 h by LPS treatment. Although the mRNA expression of *α*-S1-caisin was elevated by LPS at 24 h, its protein expression was significantly inhibited by LPS at 24 h. These observations indicated that LPS inhibits milk protein synthesis in the mammary gland. The downregulation of *Csn2* and *Lalba* transcription by LPS is possibly mediated by LPS-induced NF-*κβ* and cytokine production. It has been shown that LPS treatment of bovine MECs decreases phosphorylation of mTORC1 and its targets like ribosomal S6 kinase 1 [43], which leads to the activation of NF-*κβ*. NF-*κβ* activation has also been shown in previous mouse mastitis models [44]. NF-*κβ* activation has been shown to decrease prolactin receptor-mediated STAT5a phosphorylation, and the *Csn2* gene promoter in rodents and cattle has a consensus NF-*κβ*-binding sequence that overlaps the STAT5a binding site [45]. The decrease in phosphorylation of mTORC1 and its targets like ribosomal S6 kinase 1 can also inhibit the overall gene translation. Moreover, reduced Lalba expression can result in milk yield decrease because of its role in regulating lactose synthesis which control milk osmolality [46].

We originally hypothesized that the large recruitment of PMN to the mammary gland during mastitis could result in a local hypoxia. To test this hypothesis, we injected LPS-treated mice with the hypoxyprobe pimonidazole HCl which binds to amino acids and proteins in hypoxic regions. To our surprise, histochemical staining using FITC conjugated specific antibody to pimonidazole adducts in hypoxic cells showed that the MECs in LPS-treated gland even had a higher oxygen tension than in the PBS-treated gland. A hypoxic condition in the MECs at early lactation was demonstrated in our previous study [47]. We suspect that the improved hypoxic condition by LPS treatment observed in this study at 12 and 24 h was probably due to (1) increased blood supply to the region with inflammation and (2) reduced oxygen utilization in MECs resulting from a metabolic shift to anaerobic metabolism and reduced milk synthesis. The physiological significance of an increased oxygen tension in LPS treatment is not known. However, mRNA expression of *Hif-1α* and its target gene *Slc2a1* was not affected by LPS treatment. This was probably because the function of HIF-1*α* is mainly regulated at the protein level [48]. There was a dramatical increase of GLUT1 expression at 24 h compared to 12 h at both groups. This large increase over time may point to a potential effect of milk stasis as milk removal has not occurred in 24 h in these animals. This may result in cell differentiation towards autophagic behaviors, which promotes the expression of GLUT1 on the cell surface [49].

In summary, LPS challenge greatly induced inflammation in the mammary gland. Expression of CC3 and Bax pointed to a caspase 3 activation and potential increase in cell apoptosis by LPS, but apoptotic signals were greatly reduced by 24 h. In addition, a transient oxidative stress was increased by LPS infusion at 12 h, accompanied by enhanced expression of Nrf2-targeted antioxidative genes. Furthermore, LPS inhibited milk protein gene expression but improved hypoxic condition in the mammary gland. These observations suggest that increased cell apoptosis due to oxidative stress, reduced milk protein gene expression, and changed MEC metabolism may all contribute to the decrease in milk production during mastitis. This study provided functional insight to the mechanisms of reduced milk production during mastitis and provided possible ways to combat the reduction, such as enhancing Nrf2-antioxidant responsive element (ARE) pathway and reducing the inhibition of milk protein expression. For example, tert-butylhydroquinone, a widely used food additive and one of the most potent inducers of Nrf2 activity [40], may be tested for its potential antimastitis property.

## Figures and Tables

**Table 1:** Primers used in reverse transcription-PCR.

Gene symbol	GenBank accession number	Product length (bp)	Primer sequence (5′⟶3′)^∗^
*Csn1s1*	NM_007784.3	193	F: CCTTTCCCCTTTGGGCTTAC R: TGAGGTGGATGGAGAATGGA

*Csn2*	NM_001286022.1	330	F: CTTCAGAAGGTGAATCTCATGGG R: CAGATTAGCAAGACTGGCAAGG

*Gapdh*	NM_001289726.1	134	F: GAGCGAGACCCCACTAACATC R: GCGGAGATGATGACCCTTTT

*Lalba*	NM_010679.1	106	F: ACCAGTGGCTACGACACAC R: CGGGGAACTCACTACTTTTACAC

*Slc2a1*	NM_011400.3	361	F: CTTCGCCCTGGCCCTGCAGGAGC R: GGCACCCCCCTGCCGGAAGCCGGA

*Actb1*	NM_007393.5	334	F: TGGAATCCTGTGGCATCCA R: TAACAGTCCGCCTAGAAGCA

*Hprt*	NM_013556.2	76	F: CCCCAAAATGGTTAAGGTTGC R: AACAAAGTCTGGCCTGTATCC

*Il1b*	NM_008361.4	69	F: GCACACCCACCCTGCA R: ACCGCTTTTCCATCTTCTTCTT

*Il6*	NM_001314054.1	73	F: TCCAGAAACCGCTATGAAGTTC R: CACCAGCATCAGTCCCAAGA

*Tnfa*	NM_001278601.1	149	F: CTCCAGGCGGTGCCTATG R: GGGCCATAGAACTGATGAGAGG

*Bcl2*	NM_009741.5	205	F: GTGGTGGAGGAACTCTTCAG R: GTTCCACAAAGGCATCCCAG

*Cycs*	NM_007808.5	133	F: GAGGCAAGCATAAGACTGGA R: TACTCCATCAGGGTATCCTC

*Hif1a*	NM_001313920.1	135	F: GCTTACACACAGAAATGGCC R: AGCACCTTCCACGTTGCTGA

*Casp3*	NM_009810.3	226	F: CCTCAGAGAGACATTCATGG R: GCAGTAGTCGCCTCTGAAGA

*Casp9*	NM_001277932.1	152	F: AGTTCCCGGGTGCTGTCTAT R: GCCATGGTCTTTCTGCTCAC

*Bax*	NM_007527.3	156	F: AGTGATGGACGGGTCCGGGG R: GGCGGCTGCTCCAAGGTCAG

*Bid*	NM_007544.4	96	F: TCTGAGGTCAGCAACGGTTC R: CTCTTGGCGAGTACAGCCAG

*Ddit3*	NM_134248.2	260	F: ATGCCCATCTTCTGCTTGTCA R: CCTTGTAGTTGTGGGTCTTGT

*Nfe2l2*	NM_0010111678.2	94	F: GCAGAGACATTCCCGTTTGT R: CCTGAGGAGGAGCAGTGAAG

*Nqo1*	NM_008706.5	424	F: TCACAGGGGAGCCGAAGGACT R: GGGGTGTGGCCAATGCTGTA

*Hox1*	NM_022994.3	270	F: GCTCTATCGTGCTCGCATGA R: AATTCCCACTGCCACGGTC

*Slc7a11*	NM_011990.2	182	F: CCTGGCATTTGGACGCTACAT R: TCAGAATTGCTGTGAGCTTGCA

*Hnrnpab*	NM_001048061.1	204	F: TTTGGCGAGTTTGGGGAGATT R:GCCATACTGCTGCTGCTGATAGAC

*Stx5a*	NM_001167799.1	205	F: CGGGATCGGACCCAGGAGTTC R: CAAAGAGGGACTTGCGCTTTG

^∗^F = forward primer; R = reverse primer.

## Data Availability

The data used to support the findings of this study are available from the corresponding author upon request.
